# Natural Product Gene Clusters in the Filamentous *Nostocales* Cyanobacterium HT-58-2

**DOI:** 10.3390/life11040356

**Published:** 2021-04-18

**Authors:** Xiaohe Jin, Eric S. Miller, Jonathan S. Lindsey

**Affiliations:** 1 Department of Chemistry, North Carolina State University, Raleigh, NC 27695-8204, USA; jlindsey@ncsu.edu; 2 Department of Plant and Microbial Biology, North Carolina State University, Raleigh, NC 27695-7615, USA; esm@ncsu.edu

**Keywords:** anatoxin-a/homoanatoxin-a, hapalosin, heterocyst glycolipids, natural products, secondary metabolites, shinorine, tolypodiols, tolyporphins

## Abstract

Cyanobacteria are known as rich repositories of natural products. One cyanobacterial-microbial consortium (isolate HT-58-2) is known to produce two fundamentally new classes of natural products: the tetrapyrrole pigments tolyporphins A–R, and the diterpenoid compounds tolypodiol, 6-deoxytolypodiol, and 11-hydroxytolypodiol. The genome (7.85 Mbp) of the *Nostocales* cyanobacterium HT-58-2 was annotated previously for tetrapyrrole biosynthesis genes, which led to the identification of a putative biosynthetic gene cluster (BGC) for tolyporphins. Here, bioinformatics tools have been employed to annotate the genome more broadly in an effort to identify pathways for the biosynthesis of tolypodiols as well as other natural products. A putative BGC (15 genes) for tolypodiols has been identified. Four BGCs have been identified for the biosynthesis of other natural products. Two BGCs related to nitrogen fixation may be relevant, given the association of nitrogen stress with production of tolyporphins. The results point to the rich biosynthetic capacity of the HT-58-2 cyanobacterium beyond the production of tolyporphins and tolypodiols.

## 1. Introduction

Tolyporphins represent a class of compounds in the pigments of life family with structural features distinct from other prominent constituents, including heme, chlorophylls, bacteriochlorophylls, cobalamin, and coenzyme F_430_. Tolyporphin A, the first member of the tolyporphins family, was found in the lipophilic extract of a cyanobacterial sample. The sample, HT-58-2, was obtained from Nan Madol on the island of Pohnpei in Micronesia [1]. The discovery and identification of tolyporphin A originated with a broad screen of diverse cyanobacteria for anti-cancer activity [2]. Indeed, tolyporphin A was found to exhibit efflux pump inhibition and photocytotoxicity toward tumor cells [3,4,5].

**Figure 1:**
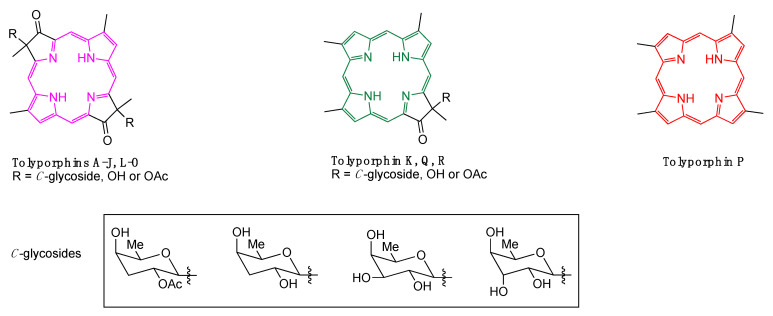
Structures of dioxobacteriochlorins (tolyporphins A–J, L–O), oxochlorins (tolyporphins K, Q, R), and a porphyrin (tolyporphin P). The π-chromophore is shown in magenta, green, and red, respectively.

An astonishing aspect of the discovery of tolyporphin A is the presence of a compound with a dioxobacteriochlorin chromophore in a cyanobacterial sample. Cyanobacteria employ chlorophyll, not bacteriochlorophyll, for photosynthesis, and the presence of compounds with a bacteriochlorin chromophore in a chlorin-based photosynthetic system is unprecedented. Over the years, fractionation of lipophilic extracts from the HT-58-2 culture has led to the identification of a family of tolyporphins, now numbering 18 (A–R) [6,7,8] (Figure 1). Most of the tolyporphins are dioxobacteriochlorins, but three are oxochlorins, and one is a porphyrin. The structural diversity suggests an in vivo role for tolyporphins as secondary metabolites in as-yet undefined defense processes. If so, this represents a fundamentally new biological function in the pigments of life family. To date, the HT-58-2 culture remains the only known producer of tolyporphins.

**Figure 2:**
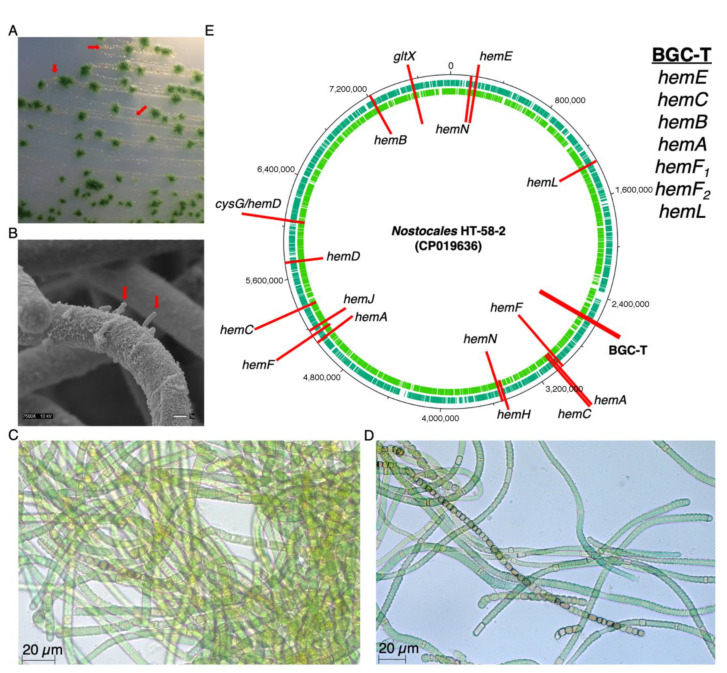
(**A**) Optical image of the HT-58-2 culture on a BG-11 agar plate shows community bacteria (red arrows) in the presence of filamentous cyanobacteria. (**B**) Scanning electron microscopy image shows community bacteria (red arrows) attached to the sheath of the filamentous bacteria. (**C**,**D**) Optical micrographs show the clumping of HT-58-2 cyanobacteria (Neofluar 40X 0.75, Zeiss) grown in BG-11 for 35 days. (**E**) Genes identified for tetrapyrrole biosynthesis pathways (termed as *hem* genes) are distributed throughout the genome of the *Nostocales* HT-58-2 (7.85 Mbp, green rings are coding sequences), except in the proposed BGC for tolyporphins (BGC-T).

To pursue questions of biosynthesis and in vivo function of tolyporphins, in late 2015 we obtained the tolyporphin-producing culture HT-58-2. Subsequent studies have revealed the following: (1) the HT-58-2 culture is dominated by a single filamentous cyanobacterium in a non-axenic cyanobacterial–microbial consortium (Figure 2A,B) [9,10]; (2) several-weeks growth under stress imparted by the deprivation of aqueous-soluble nitrate profoundly increases the production of tolyporphins, reaching a level rivalling that of chlorophyll [11]; (3) tolyporphins are present in the sheath and cell septa of the filamentous cyanobacterium, as revealed by hyperspectral confocal fluorescence imaging [12]; (4) the cyanobacterium contains a circular genome (7.85 Mbp) with genes for biosynthesis of heme, chlorophyll *a*, phycocyanobilin, and cobalamin (in part), as well as a putative biosynthetic gene cluster (here termed BGC-T) for the biosynthesis of tolyporphins (Figure 2E) [9]. Notably, BGC-T contains all genes in the core pathway (from L-glutamic acid to protoporphyrinogen IX) of tetrapyrrole biosynthesis, except *hemD*, which is located elsewhere in the genome.

In an initial report in 1992 [1], the *Nostocales* cyanobacterium HT-58-2 was categorized as *Tolypothrix nodosa* on the basis of visual inspection of the characteristic filamentous morphology (Figure 2C,D). The term “tolypothrix” refers to “a hairy ball of yarn,” whereas “nodosa” denotes the presence of nodules, which are believed to be responsible for nitrogen fixation. The availability of the sequence information enabled phylogenomic analysis, which showed the HT-58-2 cyanobacterium to be more closely aligned with the genus *Brasilonema* according to 16S rRNA [9].

**Figure 3:**
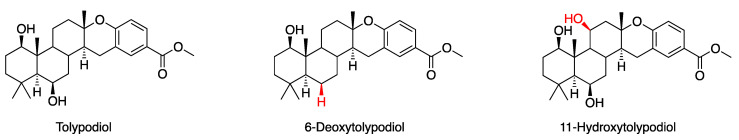
Structure of tolypodiol and two analogues isolated from cyanobacterial strain HT-58-2.

Scrutiny of the lipophilic extracts of the HT-58-2 culture has revealed other new natural products. The natural products include tolypodiol (Figure 3), the first diterpenoid compound obtained from cyanobacteria [13]. A monoacetate derivative of tolypodiol was subsequently synthesized by chemical means [13]. Both tolypodiol and the synthetic *O*-acetate derivative showed potent anti-inflammatory activity in a mouse ear edema assay. Recently, two tolypodiol analogues, 6-deoxytolypodiol and 11-hydroxytolypodiol, were also found in extracts from the HT-58-2 culture. The structures and absolute configuration of the two tolypodiol analogues were determined, and the compounds were assayed for anti-inflammatory activity [14]. Prior work [9] annotating the genome of the *Nostocales* HT-58-2 suggested numerous genes for diverse functions, but did not delve deeply into the BGCs of natural products, including those for tolypodiols.

In this paper, we report an evaluation of genes that support the biosynthesis of tolypodiols as well as other natural products, deepening the coding potential survey of the genome that was initially reported [9]. A large impetus for this work stems from two facts: (1) the in vivo functions of tolyporphins and tolypodiols are not known; and (2) the biosynthesis of tolyporphins depends markedly on environmental conditions. The production of myriad natural products by cyanobacteria can be prompted by a host of environmental stimuli [15,16]. The issue then arises as to the biosynthetic capacity of HT-58-2, i.e., what other natural products might be produced either under normal or stressed conditions?

## 2. Materials and Methods

The HT-58-2 sample was incubated in BG-11 medium as described previously [9], under continuous white light (62 µmol m^−2^ s^−1^) at 28 °C with shaking at 120 rpm. BG-11 medium provides aqueous-soluble nitrogen in the form of NaNO_3_.

Analysis of genes for the biosynthesis of natural products within the genome of *Nostocales* HT-58-2 (accession number: CP019636) was performed by the use of AntiSMASH [17], PRISM [18], and ARTS [19] with default settings. Putative BGCs for tolypodiols and nitrogen fixation were annotated and labeled manually based on protein homology searching and conserved domain analysis via the BLASTP program [20]. Individual gene or protein alignments were performed with Clustal Omega [21] and formatted with ESPript 3.0 [22].

GenBank accession numbers pertaining to the hapalosin BGC from *Fischerella* sp. PCC 9431 for protein alignment are as follows: HapA (WP_026723805), HapB (WP_035121546), HapC (WP_081656241), HapD (WP_051206727), and HapE (WP_035122279).

GenBank accession numbers for the anatoxin-a BGC from *Oscillatoria* sp. PCC 6506 are as follows: AnaJ (ACR33072), AnaA (ACR33073), AnaB (ACR33074), AnaC (ACR33075), AnaD (ACR33076), AnaE (ACR33077), AnaF (ACR33078), and AnaG (ACR33079).

GenBank accession numbers for the shinorine BGC from *Anabaena variabilis* ATCC 29,413 are as follows: Ava_3855 (ABA23460), Ava_3856 (ABA23461), Ava_3857 (ABA23462), and Ava_3858 (ABA23463).

GenBank accession numbers for the HGs BGC from *Nostoc* sp. ‘Peltigera membranacea cyanobiont’ are as follows: HetI (AGJ76607), SDR (AGJ76606), HglB (AGJ76605), PfaD (AGJ76604), HglC (AGJ76603), HglG (AGJ76602), and HglE (AGJ76601).

## 3. Results

### 3.1. Putative Tolypodiols BGC

**Figure 4:**
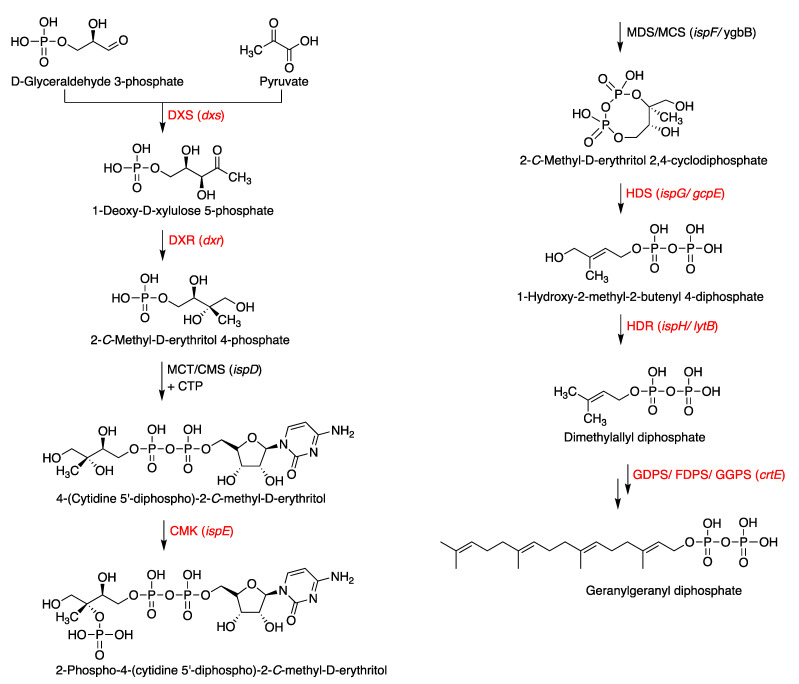
Terpenoid biosynthesis via the MEP pathway in bacteria. Proteins (genes) labeled red are present in the putative tolypodiols BGC in HT-58-2. Carboxylates and phosphates are shown in the protonated (unionized) forms.

**Figure 5:**
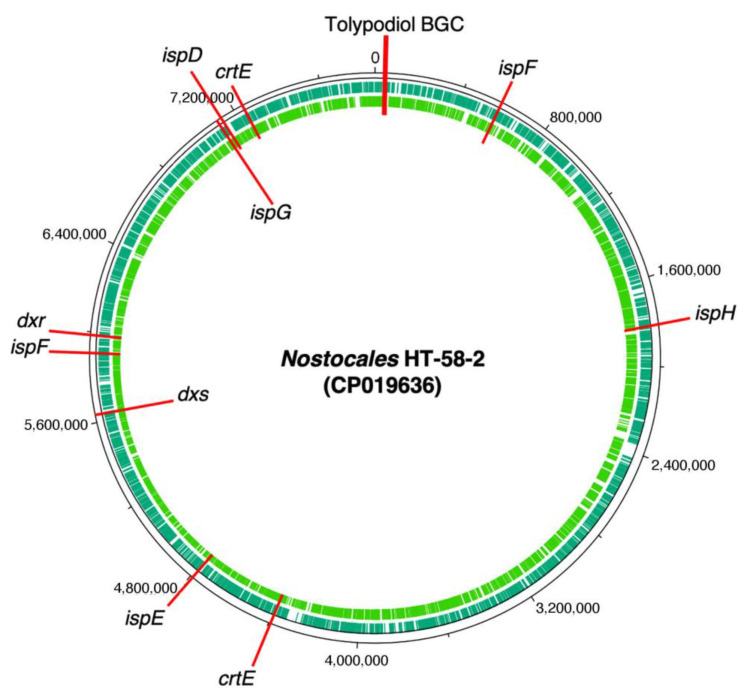
Tolypodiols BGC and distribution of genes for the MEP pathway of terpenoid biosynthesis in the genome of HT-58-2.

To pursue the biosynthesis of tolypodiols, a search for BGCs related to terpenoids in the *Nostocales* HT-58-2 was performed (hereafter in this study, “HT-58-2” will refer to the cyanobacterium unless otherwise noted). The biosynthesis of the terpenoid skeleton in cyanobacteria proceeds via a non-mevalonate pathway, termed the methylerythritol-4-phosphate (MEP) pathway. The pathway is shown in Figure 4 [23,24]. Specific genes that encode enzymes involved in the MEP pathway were annotated throughout the genome of HT-58-2; the annotation was performed manually according to protein homology. A putative BGC of ~17 kbp with genes that encode almost the entire MEP pathway was found at 38,701–55,799 bp of the HT-58-2 genome (CP019636). Two of the requisite eight genes were absent, which coded for 2-*C*-methyl-D-erythritol 4-phosphate cytidylyltransferase (MCT) and 2-*C*-methyl-D-erythritol 2,4-cyclodiphosphate synthase (MDS), corresponding to the third and fifth steps, as shown in Figure 4. The two genes missing in the BGC, *ispD* and *ispF*, occur elsewhere in the genome. The other six genes in the MEP pathway, including *dxs*, *dxr*, *ispE*, *ispG*, *ispH*, and *crtE*, appear both in the BGC and at least once elsewhere distributed in the genome of HT-58-2 (Figure 5). This situation resembles that of the putative BGC for tolyporphins, where a subset of genes within the BGC-T also appear elsewhere in the genome.

**Figure 6:**
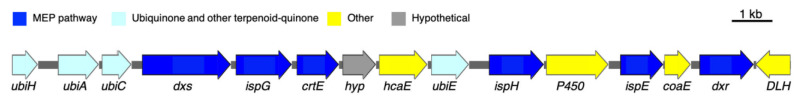
Assigned tolypodiols BGC at region 38,701–55,799 (17,099 bp) in HT-58-2. Genes encoding enzymes involved in the MEP pathway for terpenoid backbone biosynthesis are in dark blue, while those for ubiquinone/terpenoid-quinone are in light blue.

**Figure 7:**
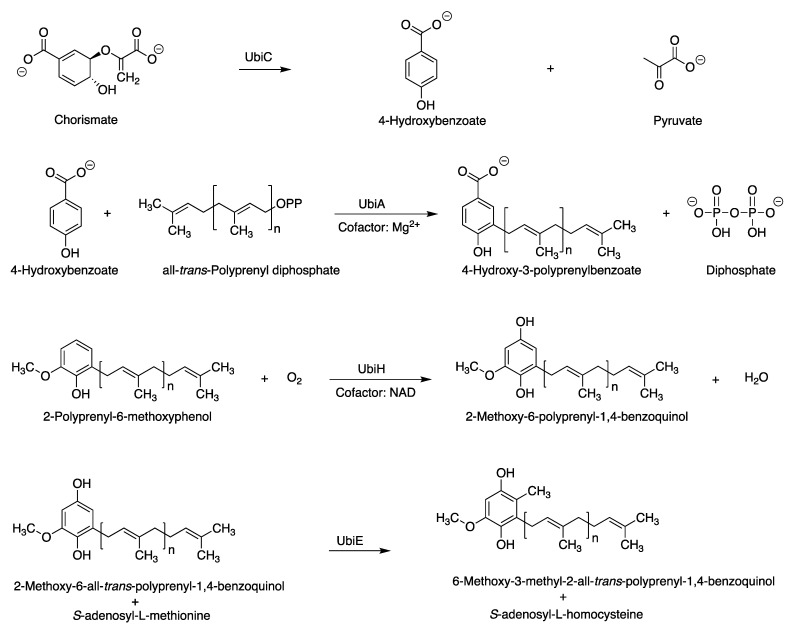
Reactions catalyzed by enzymes in the ubiquinone/terpenoid-quinone pathway. All genes for the Ubi enzymes shown here are present in the putative tolypodiols BGC in HT-58-2.

The assigned tolypodiols BGC is shown in Figure 6. In addition to the six genes for enzymes corresponding to the biosynthesis of the terpenoid backbone, four additional genes were identified that are expected to encode enzymes of the ubiquinone/terpenoid-quinone pathway. The latter pathway entails the attachment of an all-*trans*-polyprenyl unit (such as geranylgeranyl) to the 3-position of 4-hydroxybenzoate. The arene motif in the resulting polyprenyl benzoquinol has intriguing structural resemblance to the arene in tolypodiols. The corresponding enzymes UbiC, UbiA, UbiH, and UbiE are known to catalyze the reactions shown in Figure 7 [25,26,27,28]. The product from the backbone MEP pathway of terpenoid biosynthesis, geranylgeranyl diphosphate, might participate in the biosynthesis of terpenoid-quinone compounds (such as tolypodiols), given the co-clustering of *ubi*/*isp/dxr/dxs/crtE* genes. The list of aligned proteins and corresponding genes is provided in Table 1.

### 3.2. BGCs for Diverse Natural Products

**Figure 8:**
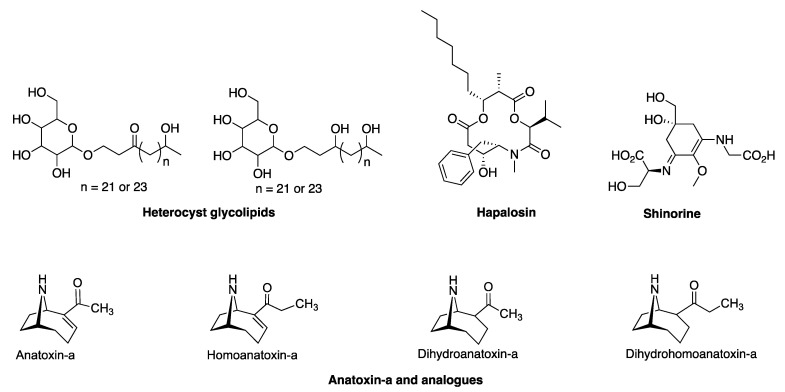
Natural products derived from putative BGCs (>50% similarity with reported ones) in HT-58-2.

In the genome of HT-58-2, eighteen clusters of genes were identified through the use of AntiSMASH (Table 2). Further analysis showed that four of the eighteen clusters (hereafter, BGCs) aligned with relatively high similarity (>50%) with known BGCs for the following compounds: (1) hapalosin; (2) anatoxin-a/homoanatoxin-a; (3) shinorine; and (4) heterocyst glycolipids (Figure 8). In-depth examination concerning the four BGCs is provided in the following section.

#### 3.2.1. Hapalosin BGC

Hapalosin is a cyclodepsipeptide that has been shown to cause the reversal of multi-drug resistance in tumor cell lines [29]. The compound has been detected from lipophilic extracts of three cyanobacterial strains: *Hapalosiphon welwitschii* UH strain IC-52-3, *Westiella intricata* UH strain HT-29-1, and *Fischerella* sp. PCC 9431. The UH strains were collected by the same team at the University of Hawaii that discovered HT-58-2 as part of a global search for novel natural products from diverse cyanobacteria [2]. Cloning and heterologous expression of the majority of the hapalosin BGC from *Fischerella* sp. PCC 9431 was recently described [30].

**Figure 9:**
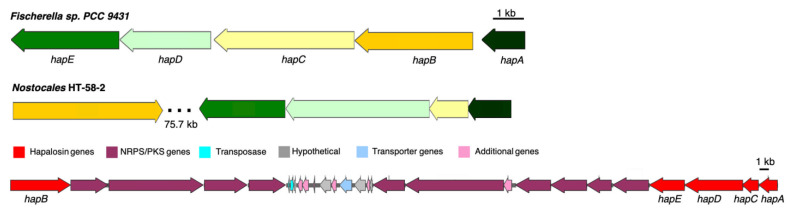
The putative hapalosin BGC identified from HT-58-2 (middle), aligned with that from *Fischerella* sp. PCC 9431 (top). Genes are color-coded to represent similarities. The lower panel shows the expansion of the 75.7 kb region of *Nostocales* HT-58-2 that contains 10 genes for NRPS/PKS systems.

In HT-58-2, a non-ribosomal peptide synthase/Type I polyketide synthase (NRPS/T1PKS)-related gene cluster was found spanning 240 kb (region nine), as shown in Table 2. More than ten T1PKS/NRPS domains are arranged in this region, which are likely involved in the biosynthesis of cyclic peptide–polyketides or lipopeptides, such as the cyclodepsipeptide hapalosin. Five genes in the BGC aligned with all five members from the hapalosin BGC from *Fischerella* sp. PCC 9431 (Figure 9) [31]. The overall percent identity between amino acid sequences of HapA-E in the two BGCs is around 50–60% with an E-value of 0.0 as given by BLASTP; the module domains of PKS and NRPS from HT-58-2 differed from those in *Fischerella* sp. PCC 9431 (Table 3). The distinct difference in sequences and modules suggests that hapalosin might not be the only product produced by this long BGC. A second group of hapalosin genes was identified (entry eight, Table 2), but the corresponding pathway appears to be incomplete.

#### 3.2.2. Anatoxin-a/Homoanatoxin-a BGC

Anatoxin-a belongs to a class of toxins produced by cyanobacteria that have been reported as threats to humans and animals [32,33]. Anatoxin-a was first isolated from *Anabaena flos-aquae* in 1977 [34], and several analogues, including homoanatoxin-a, dihydroantoxin-a, and dihydrohomoanatoxin-a, were later identified from multiple genera of cyanobacteria [35]. Known as Very Fast Death Factor (VFDF), anatoxin-a can cause tremors, paralysis, and death within a few minutes when injected into the body cavity of mice [36], and also has high acute oral toxicity [37]. Thus, monitoring water supplies for the presence of cyanobacteria that produce anatoxins has become an important issue. However, due to the limitations of many traditional means of observations such as light microscopy, it is preferable to employ genomic methods to identify cyanobacteria that have the potential to produce anatoxins.

**Figure 10:**
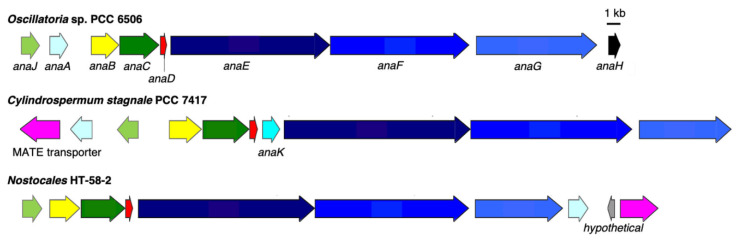
BGCs for anatoxins from *Oscillatoria* sp. PCC 6506, *Cylindrospermum stagnale* PCC 7417 and HT-58-2. Genes are color-coded to represent similarities.

**Figure 11:**
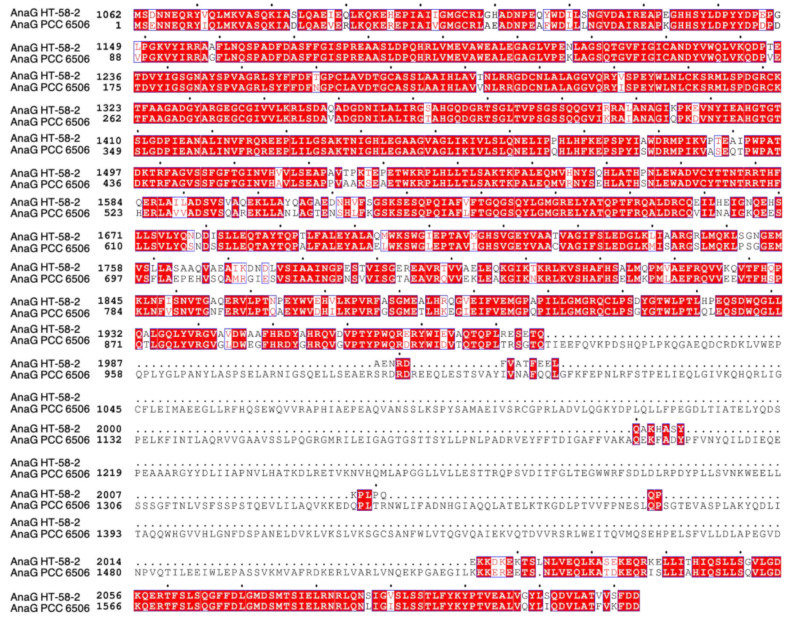
Alignment between AnaG from HT-58-2 and from *Oscillatoria* sp. PCC 6506 via ClustalW Omega. The image was generated by ESPript 3. Red highlight indicates residues of identity or high similarity, non-highlighted fonts show non-matching amino acids, and dots indicate gaps in the sequence alignment.

A putative anatoxin-a/homoanatoxin-a BGC was found between nucleotides 2,729,361 and 2,752,515 bp in the genome of HT-58-2 (Figure 10). Aligned with the reported *ana* BGC from *Oscillatoria* sp. PCC 6506 [38,39], most genes (*anaA*-*G*) share 80–90% nucleotide and amino acid identity, except for that of AnaJ with 65% aligned amino acid identity (Table 4). The main difference between the two *ana* BGCs is the position of the thioesterase gene (*anaA*). *anaA* is observed upstream of the *anaB-G* cluster in *Oscillatoria* sp. PCC 6506, versus downstream of the BGC in HT-58-2 (Figure 10). Additionally, both predicted protein functions and identified functional domains in the polyketide synthases from HT-58-2 are extremely similar to those from *Oscillatoria* sp. PCC 6506, except for that of AnaG. In particular, three module domains (KS-AT-ACP) were detected in AnaG of HT-58-2, while an additional SAM-dependent methyltransferase domain exists in *Oscillatoria* sp. PCC 6506. In Figure 11, a 599 amino acid gap can be observed in the alignment of the two respective AnaG proteins, which corresponds to the methyl transferase region on the basis of module searching. The lack of a methyltransferase in the *ana* BGC of HT-58-2 might preclude methyl extension of the acetyl group (forming the propionyl group) in the conversion of anatoxin-a and dihydroanatoxin-a to the respective homoanatoxin-a and dihydrohomoanatoxin-a, unless a methyltransferase encoded elsewhere in the genome is used.

The BGC for anatoxins found in HT-58-2 is most closely related to that identified in *Cylindrospermum stagnale* PCC 7417 (Figure 10) on the basis of BLAST results (data not shown). *C. stagnale* PCC 7417 has been reported to produce dihydroanatoxin-a, rather than anatoxin-a/homoanatoxin-a [40]. In addition to the presence and alignment of AnaA-G and AnaJ, a MATE efflux transporter is encoded by both BGCs but at distinct positions: downstream from the BGC for anatoxins in HT-58-2, while upstream in *C. stagnale* PCC 7417. The MATE efflux transporter was reported to export cyanotoxin (encoded within the saxitoxin BGC) from growing cyanobacteria [41] and might play the same role in transporting anatoxins.

#### 3.2.3. Hexose-Shinorine BGC

Cyanobacteria are known to produce pigments that afford protection against ultraviolet light. Mycosporine-like amino acids (MAAs) and scytonemins are two types of ultraviolet (UV)-absorbing compounds for protection against UV-B and UV-A radiation, respectively [42]. Shinorine is one of the ~30 MAAs earlier identified in 28 cyanobacterial strains [43] and red algae, *Porphyra umbilicalis* [44]. The structure of shinorine contains a cyclohexenimine core bearing glycine and serine substituents. Shinorine has a strong UV-absorbing potential due to a large molar absorption coefficient (ε = 28,100–50,000 M^−1^ cm^−1^) in the UV-A range [45], which generally is more penetrating than UV-B [46].

**Figure 12:**
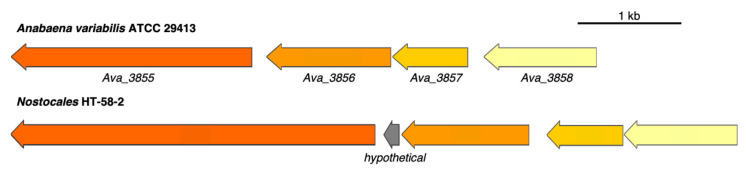
Alignment between shinorine BGCs from *A. variabilis* ATCC 29,413 and HT-58-2. Genes are color-coded to represent similarities.

A putative BGC for the biosynthesis of shinorine was identified from HT-58-2 at the region of 4,124,014–4,131,995 bp, which can be compared to the reported MAA producer *Anabaena variabilis* ATCC 29,413 [47] (Figure 12). The shinorine BGC contains four coding sequences (CDSs), with the arrangement of genes and the predicted functions of each CDS shown in Figure 12 and Table 5. Comparison can be made with an NRPS in *A. variabilis* ATCC 29,413 (Ava_3855) and in HT-58-2 (ARV60046). The latter contains an additional condensation domain, which may affect the final product derived from the shinorine BGC. Such a condensation module was also observed in NRPSs from other cyanobacterial strains, e.g., *Chlorogloeopsis fritschii* PCC 6912 [48] and *Chlorogloeopsis* sp. PCC 9212 [49]. The two *Chlorogloeopsis* strains were identified by the presence of a shinorine BGC similar to that of HT-58-2.

#### 3.2.4. Heterocyst Glycolipid BGC

**Figure 13:**
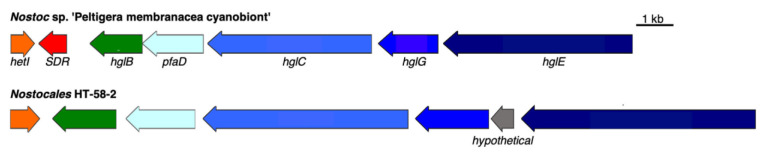
Heterocyst glycolipids BGCs from *Nostoc* sp. ‘Peltigera membranacea cyanobiont’ and HT-58-2. Genes are color-coded to represent similarities.

Heterocyst glycolipids (HGs) form a protective layer for oxygen-sensitive nitrogenase enzymes [50] in the envelope of heterocystous nitrogen-fixing cyanobacteria. The HT-58-2 cyanobacterium is filamentous and is capable of nitrogen fixation [9]; therefore, the presence of a BGC for HG biosynthesis in the genome of HT-58-2 seemed likely. Indeed, an HG BGC occurs in HT-58-2 at position 974,174–991,938 bp. Six genes therein encode proteins aligned with high similarity (>63% identity) to those of a reported BGC for HGs from *Nostoc* sp. ‘Peltigera membranacea cyanobiont’ [51]. The alignment of the two HG BGCs and the predicted product functions are shown in Figure 13 and Table 6. A short-chain dehydrogenase/reductase (SDR) is absent in the HG BGC from HT-58-2, while a carboxypeptidase regulatory-like domain protein is present between HglG and HglE. Adjacent to the assumed HG BGC are twelve photosynthesis and phycobilisome-related proteins, for which the relationship to the formation of HGs remains unknown. Overall, the putative HG BGC in HT-58-2 aligned with 85% similarity to the proteins of the *Nostoc* sp., although the effect of the absent SDR on the biosynthesis of HGs requires further study.

### 3.3. Putative BGC for Nitrogen Fixation

**Figure 14:**
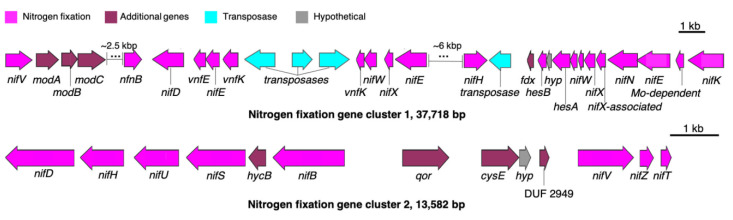
Gene clusters related to nitrogen fixation in HT-58-2. Nitrogen fixation genes are labeled in magenta, while additional genes related to ion transport or energy production are shown in maroon.

Genes for nitrogen fixation enable cyanobacteria to grow in the absence of dissolved nitrogenous compounds. The presence of a minimum set of six genes, termed *nifHDK* and *nifENB* for catalysis and biosynthesis proteins, respectively, has been reported to be essential for nitrogen fixation [52]. In HT-58-2, genes for nitrogen fixation were found to be concentrated in two regions: 4,862,885–4,900,602 bp (*nif* cluster 1) and 4,972,977–4,986,558 bp (*nif* cluster 2) (Figure 14). The former cluster contains five of the six genes (missing *nifB*), whereas the latter cluster contains three genes (missing *nifENK*); regardless, all six required genes are represented among the two clusters. The expression of the genes in the two clusters may differ under distinct growth conditions [53].

In addition to the *nif* and *vnf* genes encoding nitrogenases (the latter a vanadium-dependent nitrogenase), *modABC* genes are present in cluster 1; such genes encode proteins involved in the high affinity molybdate/tungsten uptake system. The presence of Mo-related proteins in the *nif* gene cluster 1 supports the inferred role of Mo transport in regulating nitrogen fixation by HT-58-2.

## 4. Discussion

Cyanobacteria are essential constituents of the earth’s biota and occupy diverse terrestrial and aquatic ecosystems [54,55]. Cyanobacteria provide a tremendous treasury of natural products with rich applications to serve human needs in the pharmaceutical, food, and energy industries [56]. The culture HT-58-2, which contains a filamentous cyanobacterium and several other bacteria (mostly photoheterotrophic bacteria), is known to produce two distinct classes of natural products: tolyporphins, new members of the tetrapyrrole macrocycle family; and tolypodiols, which are diterpenoids. Both sets of natural products have been subjected to initial evaluation for biological activities. Tolyporphin A was found to inhibit efflux pump activity and thereby reverse multidrug resistance (MDR) in tumor cells [1,3,4]. Tolypodiols and analogues were evaluated for anti-inflammatory properties germane to neurological disorders [13,14]. In contrast with the intriguing reports concerning bioactive properties, the biosynthetic pathways to tolyporphins and tolypodiols have been little explored, and essentially nothing is known concerning the functional roles of these natural products in the cyanobacterium and associated community bacteria.

Knowledge of the full genome of the *Nostocales* HT-58-2 enables the identification of genes or BGCs that might contribute to the biosynthesis of tolyporphins and tolypodiols. Studies of the tolyporphins BGC (BGC-T) will be reported elsewhere. Here, a BGC was examined that contains almost all genes in the MEP pathway related to the backbone biosynthesis of terpenoids. Genes co-localizing with those in the MEP pathway are *ubi* genes, which are involved in the terpenoid-quinone pathway. Additionally, the significant product of the MEP pathway, geranylgeranyl diphosphate, could react with 4-hydroxybenzoate (a product of the UbiC-catalyzed reaction derived from the common precursor chorismic acid) to afford many important aromatic compounds such as ubiquinone and vitamin K [57]. Such a BGC for tolypodiols requires further analysis to establish the complete biosynthetic steps derived from the MEP pathway. The absence of genes (*ispD* and *ispF*) corresponding to MCT and MDS proteins in the tolypodiols BGC is (1) not believed to result from an incompleteness of sequencing reads when assembling and closing the circular genome of *Nostocales* HT-58-2 [9]; and (2) is not concerning with regard to biosynthesis, because such genes are present elsewhere in the genome.

The HT-58-2 culture grows under a medium deprived of aqueous-soluble nitrate (BG-11o), consistent with a presumed nitrogen fixation capacity of heterocyst-forming cyanobacteria [11]. The production of tolyporphins is profoundly increased in the absence versus presence of soluble nitrate [11]. A minimum set of six conserved *nif* genes (*nifHDKENB*) is required for nitrogen fixation [52]. Genes for two different nitrogenases (*nif* and *vnf*) were found in two clustered regions. Nitrogen fixation in HT-58-2 is probably regulated by metal ion concentrations, because molybdate-dependent transporters (*mod*) and nitrogenases (*nif*, *vnf*) are co-localized. Further studies may probe co-relationships of gene expression for nitrogen fixation and tolyporphins production in the face of environmental stimuli.

The studies reported here suggest that HT-58-2 may be capable of producing diverse natural products beyond tolyporphins and tolypodiols. Four BGCs are aligned with relatively high similarity (above 50% similarity) with those reported for the biosynthesis of hapalosin, anatoxin-a/homoanatoxin-a, hexose-shinorine, and heterocyst glycolipids. The alignments are not complete, because a gap region occurs in the putative hapalosin BGC and at least one gene is absent in each of the BGCs. Alignment and conserved domain analysis of the protein AnaG did not reveal a means for the methylation of anatoxin-a and dihydroanatoxin-a to form the corresponding homoanatoxin-a and dihydrohomoanatoxin-a. However, the effects on product structures of absent domains, or differences in domains of individual proteins, remain unknown.

Traditional methods of discovering bioactive products from microorganisms are often limited by available cultivation conditions or purification methodologies [58]. Genome mining and bioinformatics provide complementary strategies for exploring biosynthetic pathways of metabolites. Although none of the four natural products (hapalosin, anatoxin-a/homoanatoxin-a, hexose-shinorine, and heterocyst glycolipids) has yet been isolated from the HT-58-2 culture, the identification of the putative BGCs highlights potential opportunities. The potential presence of anatoxins and analogues in particular should prompt caution in handling the HT-58-2 cultures and extracts thereof, given their known physiological effects. Moreover, biological assays with crude extracts must be cautiously interpreted given the possible presence of numerous bioactive natural products. Identification of putative BGCs enables further work to address issues concerning possible evolutionary origin. Taken together, the putative BGCs described in this study establish a framework for investigation of the biosynthesis of tolypodiols and other natural products, as well as nitrogen fixation and regulation in the HT-58-2 cyanobacterial–bacterial consortium.

## Figures and Tables

**Table 1:** Aligned proteins from the putative tolypodiols BGC in HT-58-2 ^a^.

HT-58-2 Accession	Aligned Protein (Gene)
WP_015211021	2-polyprenyl-6-methoxyphenol hydroxylase (*ubiH*) ^b^
ARV62772	4-hydroxybenzoate polyprenyltransferase (*ubiA*)
ARV57253	chorismate lyase (*ubiC*)
ARV57254	1-deoxy-D-xylulose-5-phosphate synthase (*dxs*)
ARV57255	1-hydroxy-2-methyl-2-(E)-butenyl 4-diphosphate synthase (*ispG*)
ARV62773	geranylgeranyl pyrophosphate synthase (*crtE)*
ARV57256	hypothetical protein
ARV57257	phenylpropionate dioxygenase or ring-hydroxylating dioxygenase (*hcaE*)
ARV57258	ubiquinone/menaquinone biosynthesis *C*-methylase (*ubiE*)
ARV57259	4-Hydroxy-3-methylbut-2-enyl diphosphate reductase (*ispH*)
ARV57260	cytochrome P450
ARV62774	4-diphosphocytidyl-2C-methyl-D-erythritol kinase (*ispE*)
ARV62775	dephospho-CoA kinase (*coaE*)
ARV57261	1-deoxy-D-xylulose 5-phosphate reductoisomerase (*dxr*)
ARV57262	Dienelactone hydrolase

^a^ All E-values of aligned proteins are <2.8e-10. ^b^ E-value is 1.6e-04.

**Table 2:** Putative BGCs for natural products identified in HT-58-2 by AntiSMASH.

No.	Region (bp)	Length (nt)	Type	Similar Known Cluster
1	308,169–329,626	21,458	lassopeptide	
2	344,287–393,016	49,759	NRPS ^a^	vioprolides, xenoamicins, cyanopeptin
3	855,604–866,222	10,619	RiPP-like ^b^	
4 *	958,778–1,011,938	53,161	hglE-KS ^c^, T1PKS ^d^	heterocyst glycolipids
5	1,080,296–1,132,473	52,178	T1PKS	carbamidocyclophanes
6	1,225,583–1,267,343	41,761	phosphonate, terpene	
7	1,620,824–1,643,303	22,480	lassopeptide	
8	1,748,080–1,798,143	50,064	NRPS	hapalosin
9 *	2,268,098–2,291,911	240,666	NRPS, T1PKS	hapalosin
10	2,303,699–2,508,763	20,507	NRPS, T1PKS	nostopeptolide A2
11 *	2,683,411–2,768,967	85,557	T1PKS	anatoxin-a
12	3,624,228–3,644,311	20,084	terpene	
13	3,794,851–3,850,264	55,414	T1PKS	chondrochloren A
14	4,005,987–4,016,220	10,234	bacteriocin	
15 *	4,105,987–4,016220	42,348	NRPS	hexose-palythine-serine
16	4,214,166–4,256,226	41,891	bacteriocin	
17	4,262,518–4,353,871	91,354	NRPS, lanthipeptide	nostopeptolide A2
18	5,824,405–5,845,337	20,933	terpene	

* Clusters of known BGCs wherein >4 core genes were identified with high similarity. ^a^ Non-ribosomal peptide synthetase cluster. ^b^ Other unspecified ribosomally synthesized and post-translationally modified peptide product clusters. ^c^ Heterocyst glycolipid synthase-like polyketide synthase (PKS). ^d^ Type I polyketide synthase (PKS).

**Table 3:** Alignment between proteins of the putative HT-58-2 hapalosin BGC and those from *Fisherella* sp. PCC 9431.

HT-58-2 Accession	Aligned Protein	E-Value	% Identity *
ARV58870	HapA, AMP-dependent synthetase	0.0	60.2%
ARV58846	HapB, polyketide synthetase	0.0	51.6%
ARV58869	HapC, non-ribosomal peptide synthetase	0.0	58.8%
ARV58868	HapD, non-ribosomal peptide synthetase	0.0	63.6%
ARV62947	HapE, polyketide synthetase	0.0	65.3%

* The percent identity included >75% of aligned protein length from the two BGCs, except for HapD (42%).

**Table 4:** Alignment between proteins of the putative HT-58-2 anatoxin-a BGC and those from *Oscillatoria* sp. PCC 6506.

HT-58-2 Accession	Aligned Protein	E-Value	% Identity *
ARV59080	AnaJ, cyclase	0.0	69.0%
ARV59087	AnaA, thioesterase	3e-157	82.8%
ARV59081	AnaB, acyl-CoA dehydrogenase	0.0	87.9%
ARV59082	AnaC, proline adenylation	0.0	87.7%
ARV59083	AnaD, acyl carrier protein	2e-51	87.4%
ARV59084	AnaE, polyketide synthase	0.0	86.7%
ARV59085	AnaF, polyketide synthase	0.0	82.2%
ARV59086	AnaG, polyketide synthase	0.0	83.9%

* The percent identity included >90% of aligned protein length from the two anatoxin-a BGCs.

**Table 5:** Alignment between proteins of the putative HT-58-2 shinorine BGC and those from *A. variabilis* ATCC 29413.

HT-58-2 Accession	Aligned Protein	E-Value	% Identity *
ARV60049	3-dehydroquinate synthase	3e-149	78.7%
ARV60048	SAM-dependent methyltransferase	1e-109	74.2%
ARV60047	ATP-grasp enzyme	1e-215	79.0%
ARV60046	NRPS	0.0	72.9%

* The percent identity included >66% of aligned protein length from the two shinorine BGCs.

**Table 6:** Alignment between proteins of the putative HT-58-2 heterocyst glycolipids BGC and those from *Nostoc* sp.

HT-58-2 Accession	Aligned Protein	E-Value	%Identity *
ARV57925	HetI, 4′-phosphopantetheinyl transferase	1e-125	71.2%
ARV57926	HglB, thioester reductase	0.0	73.0%
ARV62843	PfaD, polyunsaturated fatty acid/polyketide biosynthesis protein	0.0	79.5%
ARV57927	HglC, beta-ketoacyl synthase	0.0	63.6%
ARV57928	HglG, polyketide synthase	0.0	65.3%
ARV57929	carboxypeptidase regulatory protein	N/A	N/A
ARV57930	HglE, polyketide synthase	0.0	66.8%

* The percent identity included >97% of aligned protein length from the two HG BGCs.

## Data Availability

Data are available from the corresponding author on reasonable request.
